# Women's voices: the lived experience of HIV-positive women in the postpartum period at the University of Gondar comprehensive specialized referral hospital, Ethiopia: a phenomenological study

**DOI:** 10.1186/s12905-023-02470-5

**Published:** 2023-06-21

**Authors:** Workie Zemene Worku, Telake Azale, Tadesse Awoke Ayele, Dawit Kassahun Mekonnen

**Affiliations:** 1 Department of Community Health Nursing, School of Nursing, College of Medicine and Health Sciences, University of Gondar, Gondar, Ethiopia; 2 Department of Health Education and Behavioral Sciences, Institute of Public Health, College of Medicine and Health Sciences, University of Gondar, Gondar, Ethiopia; 3 Department of Epidemiology and Biostatistics, Institute of Public Health, College of Medicine and Health Sciences, University of Gondar, Gondar, Ethiopia; 4 Department of Gynaecology and Obstetrics, School of Medicine, College of Medicine and Health Sciences, University of Gondar, Gondar, Ethiopia

**Keywords:** Lived experience, Pregnant women, HIV infection, Amhara Region, Ethiopia

## Abstract

**Background:**

Pregnancy and the postpartum period are incredibly challenging for women living with the Human Immune Deficiency Virus (HIV) due to the multidimensional influence of HIV infection. These women experience the challenges of navigating pregnancy and motherhood while living with HIV. It is poorly understood how women living with HIV (WLWH) experience pregnancy and postpartum. Therefore, the current study aimed to explore the lived experience of pregnancy and postpartum among women living with HIV.

**Methods:**

A phenomenological qualitative study design was employed. A semi-structured, interview guide was used to conduct in-depth interviews with HIV-positive postpartum women from April to May 2022. All interviews were recorded using a voice recorder and note was taken. The collected data were transcribed and translated into English for analysis. Inductive thematic analysis was applied to analyze the data.

**Results:**

Six main themes about the lived experience of women living with HIV were identified: (1) Fear and distress related to maternal and child Health, (2) HIV status self-disclosure dilemma, (3) Courage and commitment of HIV-positive women to prevent HIV, (4) Challenges faced and coping mechanisms used by HIV-positive women, (5) Health care providers and HIV-positive women interaction, and (6) Solution from their voices.

**Conclusion:**

HIV infection also has a multidimensional impact on women’s life during pregnancy and postpartum. The findings of this study improve our understanding of the lived experience of HIV-positive women while pregnant and in the postpartum period. These women's descriptions showed that they have been experiencing various challenges that are not often addressed in antenatal clinics, such as distress and uncertainties related to vertical transmission of HIV. These women need specialized support and all-encompassing care to have a healthy pregnancy and postpartum period. Moreover, it is essential to understand the circumstances of women's lives, their relationships with others, and their decision-making processes. Healthcare professionals and other responsible bodies working with women living with HIV should encourage and support these mothers to appraise and maintain their commitment to protecting their children from acquiring HIV infection and maintaining their Health to the maximum to raise their children.

**Supplementary Information:**

The online version contains supplementary material available at 10.1186/s12905-023-02470-5.

## Plain Language Summary

Worldwide, a significant number of people have contracted HIV infection, of them; women of reproductive age constitute more than half of the overall infected population. With the development of antiretroviral drugs, coupled with better care for individuals with sero-positive status, a considerable number of HIV positive women become pregnant each year globally. Yet, the multifaceted effects of HIV make their lived experiences of pregnancy and postpartum difficult and full of doubts and concerns. However, there is a dearth of studies on the lived experience of HIV positive women during pregnancy and postpartum in Ethiopia.

This qualitative study was conducted to explore the lived experience of HIV positive women in the postpartum period. A total of 13 HIV positive postpartum women at the University of Gondar Comprehensive Specialized Hospital were recruited for the study. Data were collected using one-on-one in-depth interview.

Six themes were emerged about the lived experience of HIV positive women throughout their pregnancy and postpartum time. These include: Fear and distress related to maternal and child Health, HIV status self-disclosure dilemma, courage and commitment of HIV-positive women to prevent HIV, challenges faced and coping mechanisms used by HIV-positive women, Health care provider's and HIV positive women interaction, and Solution from their voices.

A specialized, individualized, and all-encompassing care should be designed for these women to promote their overall wellbeing and have a healthy pregnancy as well as raise their children with a minimal uncertainty.

**Supplementary Information:**

The online version contains supplementary material available at 10.1186/s12905-023-02470-5.

## Background

A significant number of women and girls globally have continued to experience the devastating effects of HIV infection since the virus was first detected. An estimated 38.4 million people were living with HIV globally; of this, 1.5 million people were infected with Human Immune Deficiency Virus (HIV) in the year 2021 [1]. On a global scale, women constitute 54% of all HIV-positive people, with an estimated 19.3 million women having the virus by 2021. Women and girls comprise 63% of all new HIV infections in sub-Saharan Africa in 2021 [1].

HIV remains a serious public health concern for Ethiopia's working and reproductive populations despite the country's impressive accomplishments in preventing and controlling the pandemic [2, 3]. The Joint United Nations Program on HIV/AIDS (UNAIDS) 2020 estimates that 620,000 HIV-positive people are living in Ethiopia. Of these, 360,000 were women of childbearing age, with 8,900 adults newly infected with the virus [4]. Ethiopia is among the top 25 countries worldwide regarding new HIV infections [3].

Considerable efforts have been made to decrease the impact of HIV infection on people's life since its spread around the world as an epidemic. The introduction of antiretroviral medication made the disease condition potentially manageable. As a result, there was a noticeable decrease in the mortality and morbidity linked to HIV and AIDS. Consequently, there has been a 48% decrease in AIDS-related mortality since 2003. However, the virus's presence still calls for a reassessment of the lives of women infected with it [1, 5–7].

The Joint United Nations Program on HIV/AIDS launched a global plan in 2011 to prevent new HIV infections in children born from HIV-positive mothers and keep mothers alive. The main objective of this initiative is to reach pregnant women living with HIV and their children from the time of pregnancy until the mother quits breastfeeding [8, 9]. According to this approach, regardless of the CD4 count or clinical stage, lifelong triple antiretroviral therapy (ART) must be provided to pregnant women as soon as their HIV positivity is confirmed [10].

Ethiopia joined the Global Plan in June 2011 to prevent new HIV infections in children and ensure the survival of their mothers [11, 12]. Many women living with HIV desire to have children because women living with HIV think that being pregnant and becoming a mother enhances their self-worth [13]. Besides, the availability of ART and other pertinent interventions during pregnancy, labor, and the postpartum period helps women to develop hopes of getting an HIV-free child. Evidence has also shown that ART can lead to a dramatic decrease in mother-to-child transmission of HIV (MTCT). It can be decreased to less than 5% by providing all necessary interventions in the PMTCT services [14–16]. This results in over 2 million HIV-positive women giving birth yearly worldwide [17]. Ninety percent of all HIV-related pregnancies worldwide occur in Sub-Saharan Africa [18]. In 2021, 81% of HIV-positive pregnant women received ART to prevent mother-to-child transmission [19, 20].

Motherhood is the process of becoming a mother, and it begins when a woman decides to get pregnant. The time of motherhood encompasses the period of pregnancy, the gap or time between pregnancies, and the rest of a woman's life [21]. In this regard, seeking pregnancy to see the offspring and obtaining a desired family family size is a dream of couples and a fundamental right to reproduction for all people [22]. Pregnancy as a developmental phase involves physiological and psychological changes that are normal and tolerable to a certain extent. At any point of the pregnancy, however, the physical discomfort of the expectation of giving birth and becoming a parent typically creates psychological distress in some pregnant mothers [23, 24].

For women living with HIV, the burden becomes double since they deal with the effects of HIV in their daily lives. These effects are amplified during pregnancy, childbirth, and postpartum due to women's dual roles as patients and mothers [6, 21, 25–27]. Despite the success of combination antiretroviral therapy (cART), which has helped to normalize pregnancy relatively, the process of pregnancy and labor, the postpartum process, becomes intense. Stressors associated with HIV, such as guilt feelings, worries, and uncertainty about the possibility of HIV transmission to the baby, about the mother's health, and about meeting the specific needs of their newborns, in addition to adjusting to motherhood as usual, disturb the physiologic and psychosocial homeostasis [16, 21, 26, 27].

Evidence shows that most women living with HIV are at risk and frequently face psychosocial challenges during pregnancy, childbirth, and postpartum [7, 13, 16, 28, 29]. These impacts heighten their sense of isolation and prevent them from engaging with their social communities, which can further deteriorate their experience during pregnancy and postpartum [27, 30].

Women living with HIV reportedly use various coping mechanisms to deal with the wide range of experiences they encounter during pregnancy and after giving birth [28, 31]. Given the high risk of HIV transmission from mother to child, women living with HIV have access to various clinical interventions during pregnancy, childbirth, and postpartum [32]. Despite, women with HIV still experience various medical and psychosocial challenges during pregnancy and postpartum. These challenges are severe for those who live in resource-poor countries because pregnancy and the postpartum period add additional pressures related to partner dynamics, societal expectations, and healthcare requirements [21, 32].

However, the realities of their lived experience as a mother living with HIV are still little understood. This is particularly crucial because women's sexual and reproductive health plays a vital role in ensuring their wellbeing and the well-being of their partners and children. Yet, there is a shortage of studies in general and in Ethiopia in particular on the lived experience of HIV-positive women throughout pregnancy, childbirth, and postpartum. Little is known about how HIV affects women's experiences throughout pregnancy, labor, and postpartum. Therefore, it is crucial to reveal how HIV-positive women deal with pregnancy and postpartum since these aspects of their life receive less attention [16]. The current study aimed to generate an interpretive narrative on the lived experience of HIV-positive women during pregnancy and in the postpartum period. The findings will help health professionals, program planners as well as nongovernmental organizations working on combating HIV and related issues to develop essential and applicable interventions, prevention as well as planning policies and practices to improve the women's life experience during pregnancy and in the postpartum.

## Methods

### Study design

A qualitative descriptive phenomenological study design was employed to explore the lived experience of pregnancy and the postpartum period among women living with HIV from April to May 2022. Phenomenology serves as a conceptual foundation that enables researchers to identify and characterize the essence of an event from the participant's point of view [33]. The main objective of phenomenological research design is to uncover the reality in people's descriptions of how they experience the world, to record emotions and lived experiences, and to produce in-depth reports of a specific occurrence as accurately as possible. These descriptions should be based on meaningful and significant lived experiences of the phenomenon [34, 35]. This study used semi-structured, in-depth interviews conceptually informed by phenomenological study design.

### Participant selection

The study was conducted on postpartum women living with HIV attending postnatal care (PNC) at the PMTCT clinic of the University of Gondar Comprehensive Specialized Hospital. The PMTCT encompasses a series of services offered to HIV-positive women, including antenatal care, HIV testing during pregnancy, antiretroviral therapy (ART), safe delivery techniques, optimal newborn feeding, and HIV testing of the child [14]. Aligned with the study goal, the inclusion criteria to participate in the study was a woman with a diagnosis of HIV before the current child and was currently in the postpartum period.

### Sampling

Study participants for in-depth interviews (IDIs) were selected using a purposive sampling method. The participants were chosen when they visited the clinic to get PMTCT services after consulting the health professionals working at the PMTCT clinic. Then they were approached by the principal investigator. Purposive sampling has the advantage of choosing individuals with a wealth of information, allowing the researcher to analyze the topic under study in greater depth and providing insights and in-depth understanding as opposed to making empirical generalizations [36].

### Sample size

Thirteen postpartum women living with HIV were interviewed which was determined by the information saturation criterion., Data saturation indicates that repeated interviews produce information that is already sufficiently rich or no new information emerges [37].

### Study setting

The study was carried out at the University of Gondar Comprehensive Specialized Hospital, located in Gondar town, Central Gondar Zone, North West Ethiopia, 727 km from Addis Ababa [38]. This facility offers focused ANC services and has a separate ART and PMTCT clinic. The Ethiopian government began implementing Option B + (initiation of antiretroviral medication for all expectant mothers and continued throughout their lives, regardless of CD4 count) in 2013 [12]. Since then, the service has been available in all health facilities at no cost.

### The setting of the data collection

The data were collected in one of the offices close to the PMTCT clinic where HIV-positive postpartum women receive clinical follow-up. The interviews took place in a secure and private setting with little background noise and voice to retain the quality of the recording and allow open dialogue. This was done to ensure the women's privacy, maintain confidentiality, and ensure the accuracy of the reports.

### Data collection tools

#### Instrument

Semi-structured interview guide was used to conduct face-to-face one–on–one in-depth interviews with participants. We developed the interview guide by reviewing a range of literature in the field. It was initially prepared in English and then translated to Amharic (participants' native language) to ensure the questions' consistency and extract details of the data through probes. The interview guide was developed to capture the lived experiences of HIV-positive women, from HIV diagnosis, during pregnancy to the postpartum period. Inputs were obtained from reproductive medicine, public Health, and mental health experts. The in-depth interviews lasted 40 min on average. The principal investigator, a nurse, and reproductive health professional conducted the interview. The interview guide is presented at the end of the reference of this manuscript.

The audio recording along with note taking was used to collect the data.

### Data saturation

In the current study, the term "saturation" refers to the phase of data collection when new interviews yielded little to no additional information relevant to the research issue. According to the available literature, a minimum of 12 interviews are usually required [37, 39]. A point of saturation was reached for our research question of women's lived experiences during the postpartum period.

### Data analysis

Demographic and obstetric information for each participant was collected using the interview. With the participants' permission, voice recorders captured all information. The recorded interviews were transcribed verbatim and then translated into English to conduct a thematic analysis. Inductive thematic analysis was used to analyze the data. The purpose of thematic analysis is to comprehend patterns of meanings from data on lived experiences. Thematic analysis is a technique for finding, examining, and reporting patterns (themes) within data. Thematic analysis is a theoretically flexible method for analyzing qualitative data that looks for themes or patterns since a theoretical framework does not confine it. Thematic analysis can, therefore, be a technique that reflects reality and unravels the surface of reality [40, 41]. The data analysis was carried out by the recommendations for thematic analysis by Virginia Braun and Victoria Clarke [40, 41]. The analysis process was carried out using the six main phases stated below.

### Phase 1: familiarizing with the data

The research team immerses in the data to the extent that we are familiar with the depth and breadth of the content through listening to the audio recording, reading the note and transcriptions, and rereading the transcripts, as this phase is where the basis of the analysis is laid. The study team looked for patterns and significance in the data during this procedure.

### Phase 2: generating initial codes

Initial codes were generated from the data during this step after a codebook for interviews was designed by the research team using inductive thematic analysis. Codes refer to "the most fundamental segment, or element, of the raw data or information that can be assessed in a meaningful way addressing the phenomena" [40, 41].

### Phase 3: searching for themes

A theme is a significant aspect of the data related to the research topic and denotes a level of patterning or significance within the data set Field [40, 41]. In this stage, themes rather than codes serve as a new focal point for the study. The research team organized the codes found across the data set to create broad themes. Following this, we gathered all the relevant coded data extracts within the themes and sub-themes.

### Phase 4: reviewing themes

In this phase, the research team refined the themes that emerged in phase three by reviewing them, reading all the codes assigned to each theme, and determining whether they fit together to form a logical pattern. Then, as additional codes were discovered as emergent themes, we added them to the codebook and made any necessary adjustments.

### Phase 5: defining and naming themes

After having a satisfactory thematic map of our data, we designated names for each theme. The identified themes include: (1) Fear and distress related to maternal and child Health, (2) HIV status self-disclosure dilemma, (3) Courage and commitment of HIV-positive women to prevent HIV, (4) Challenges faced and coping mechanisms used by HIV-positive women, (5) Health care provider's and HIV positive women interaction and (6) HIV positive women's recommendations to stop new HIV infection and minimize stigma and discrimination. Finally, we began defining the core of each theme's content, and the analysis was based on these themes by the research questions. The data analysis stayed close to the participant descriptions despite the researchers' prior knowledge and experience.

### Phase 6: producing the report

The final analysis and report writing were done during this stage. The written version of the report reflects the lived experiences of HIV-positive women since they initially discovered their HIV-positive status during pregnancy and the postpartum period. A clear, cohesive, logical, non-repetitive, and engaging narrative is presented. We included numerous quotes from the participants' descriptions to illuminate the key findings.

### Trustworthiness

The following actions were taken to ensure the trustworthiness of this study. Before conducting the interview, the interview guide was first developed by reviewing a large body of literature. Experts evaluated it, including a professor with extensive experience in qualitative research and specialists in reproductive health medicine, nursing, and public Health. The interview guide was checked whether it was culturally sensitive. The principal investigator conducted the interviews using the local language, Amharic. The researcher interviewed following medical ethics, upholding the ethical standards in a private, secure setting. Credibility of this study was maintained through the following measures. The research team rigorously examined the data and presented the findings, which were fully based on participant descriptions rather than potential researcher biases. This is confirmed by the final report, which also uses the participants' words to support the study's conformability/reflexivity. Furthermore, the thorough data analysis process that shows how the thematic analysis was carried out using a conceptual map ensures the themes' internal coherence and validity and maintains the study's credibility. Along with this, the study's credibility was maintained by keeping in touch with participants for an extended period of time as they sought out PMTCT services. Moreover, to increase the credibility of the qualitative studies member checking was done by providing the transcription for three participants in order to verify that the transcription and summary of the interview were what they supplied or compatable with their experiences. This study provides a through description of the demographics characteristics and study setting of the study participants. Besides, a range of lived experiences from their narratives were well presented in the result section, this could help the reader to design interventions for similar population, by doing this we ensure the transferability. Giving a thorough explanation of the research methodologies, which included explicitly stating the study's aim, describing the selection process and participants' motivations, describing the data gathering process and the duration of the data collection, describing the process of data reduction or transformation and ready for the analysis, and describing how the results were interpreted and presented, helped to ensure the dependability of this study. The number of participants was determined by the saturation of information.

## Findings

### Socio-demographic and obstetric characteristics of the study participants

A total of 13 HIV-positive postpartum women attending the PMTCT clinic of the University of Gondar Comprehensive Specialized Hospital participated in the in-depth interviews. Participants' ages range from 25 to 40 years, with a mean age of 33. Eleven out of thirteen women were married; of those, only three women's husbands were HIV-negative. The majority (eleven out of thirteen women) reported that their birth was unplanned. Around half of the women said that they never went to school. Six of the participants admitted that they first discovered they had HIV while seeking health care at the hospital, while seven of the participants have been taking ART for over ten years (minimum = 2 years, maximum = 18 years). Almost all of the participants disclosed their HIV-positive status to their spouses. The details of the socio-demographic and obstetric characteristics of the study participants are shown in Table 1.

**Table 1:** Socio-demographic and obstetrics characteristics of the study participants, Northwest Ethiopia, 2022 (*n* = 13)

No	ID	Age	Residence	Marital status	Occupation	Educational level	Birth plan	Child spacing in years	HIV diagnosis context	Duration of living with HIV in years	Duration of ART in years	Spouse's HIV status	Breastfeeding status	Disclosure status
1	P01	34	Urban	Single	Daily labourer	Primary school	Not planned	7	On labour	14	14	No spouse	Breastfeed	Not disclosed
2	P02	40	Urban	Single	Prostitute	Never went to school	Not planned	19	During severe illness	19	18	No spouse	Breastfeed	Disclosed to the public
3	P03	32	Urban	Married	Housewife	Secondary school completed	Not planned	5	During an illness	12	12	HIV positive	Breastfeed	Disclosed only for my husband and my family
4	P04	32	Urban	Married	Housewife	Secondary school completed	Planned	5	during pregnancy	8	8	HIV positive	Breastfeed	Disclosed only for my husband
5	P05	36	Urban	Married	Merchant	Secondary school completed	Not planned	2	During an illness	4	4	HIV positive	Breastfeed	Disclosed only for my husband
6	P06	29	Rural	Married	Housewife	Never went to school	Not planned	9	During an illness	12	12	HIV positive	Breastfeed	Disclosed only for my husband
7	P07	38	Rural	Married	Daily laborer	Never went to school	Not planned	3	During an illness	8	8	HIV negative	Breastfeed	Disclosed to my husband, my family, and the public
8	P08	36	Rural	Married	Daily laborer	Primary school completed	Not planned	4	During pregnancy	11	11	HIV negative	Breastfeed	Disclosed forto husband, my family, and the public
9	P09	25	Rural	Married	Housewife	Primary school completed	Planned	My 1^st^ child	During pregnancy	2	2	HIV negative	Breastfeed	Disclosed only for my husband
10	P010	27	Urban	Married	Housewife	Never went to school	Not planned	2	On labour	3	3	HIV positive	Breastfeed	Disclosed only for my husband and his family to know
11	P011	32	Urban	Married	Housewife	Secondary school completed	Not planned	3	During pregnancy	12	12	HIV positive	Breastfeed	Disclosed only for my husband
12	P012	38	Urban	Married	Merchant	College diploma	not planned	14	VCT	11	11	HIV positive	Breastfeed	Disclosed to my husband, my family, and the public
13	P013	39	Rural	Married	Daily labourer	Never went to school	not planned	10	VCT	10	10	HIV positive	Breastfeed	Disclosed to husband, my family, and, to public

*VCT* Voluntary Counseling and testing

### Themes and sub-themes

**Fig. 1:**
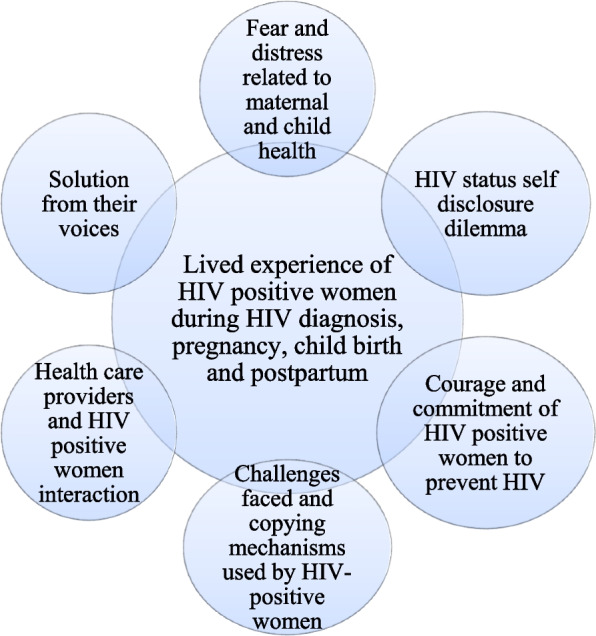
Visual framework of the lived experience of HIV positive postpartum women in Ethiopia

During the data analysis, six main themes about the lived experience of women living with HIV were identified. These include (1) Fear and distress related to maternal and child Health, (2) HIV status self-disclosure dilemma, (3) Courage and commitment of HIV-positive women to prevent HIV, (4) Challenges faced and coping mechanisms used by HIV-positive women, (5) Health care provider's and HIV positive women interaction and (6) Solution from their voices shown in Fig. 1.

### Fear and distress related to maternal and child Health

Nearly all of the women reported that they had undergone various stages of emotional reactions, mainly fear and distress, throughout their lives, primarily after receiving their first HIV test results while pregnant and in the postpartum period.

### Fear and distress upon HIV diagnosis

Although the women in this study responded to the HIV diagnosis in different ways following the discovery of their initial HIV-positive test results, all study participants claimed to have been shocked and distressed, and some had long-term health effects. For some of the participants, the HIV-positive diagnosis was unexpected. In contrast, for others, it was anticipated and believed it would happen eventually due to their risk-taking behavior. Some of the participants had been tested for HIV as they came to seek health care. The participants' narratives provide the following information regarding how they initially felt after discovering they were HIV positive for the first time. For instance, a 34-year-old woman who has been living with HIV for 14 years stated:

*"I found out that I was HIV positive while giving birth, and at that time, I was upset, shocked, and about to commit suicide, but the doctor came and saved my life.",*

Another participant disclosed:

"*I found out that I was HIV positive when I was hospitalized due to a severe illness; at that time, I was crying so much that I thought I would soon pass away from the illness,"* a 40-year-old woman living with HIV for 19 years.

A 25-year-old woman who has been living with the virus for two years stated:

*"I found out that I was HIV positive when I was pregnant. When I heard that I was HIV positive, I was very distressed. I even didn't want the baby to be born. I just stated that the fetus needed to be aborted; I also decided to attempt the abortion on my own. I saw no positive results, and I became quite anxious. On the same occasion, my husband checked his HIV status and assured me that I would be fine. He also promised never to leave me alone and to always be by my side. In addition, the doctors gave me a lot of advice, which helped me to give birth to the baby gradually."*

### Fear and distress during pregnancy, labor, and postpartum

Women experienced various emotions throughout pregnancy, labor, and postpartum, including happiness and uncertainties. It is well recognized that women who have HIV experience pregnancy, labor, and the postpartum period differently from women who do not have HIV.

All participants experienced uncertainties, fear, and distress during pregnancy, labor, and postpartum. The main source of such psychological difficulties was the HIV status of the child, contingent on the possibility of mother-to-child HIV transmission during pregnancy, labor, and postpartum while breastfeeding. Lack of financial resources has given the mothers no other option, such as formula feeding. Anecdotes from the participants reveal the psychological states at this phase.

*"I was overwhelmingly worried about my daughter and if she would acquire HIV during pregnancy, labor, and through my breast milk. This worry kept me woke up at night for many nights; being HIV positive and breastfeeding a child is more painful than labor pain and more depressive than being in a dark place. I'm also feeling bad since she might get punished for my mistakes. All these emotions will persist until my daughter's HIV status is confirmed,"* a 34-year-old *woman, living with HIV for 14 years."*

*A 32-year-old woman who has been living with the virus for seven years revealed:*

*"I am afraid that the virus will be transmitted to my baby that he doesn't know anything about. No matter how careful you are, there is still danger. I am terrified of whether the virus will be transmitted through pregnancy, delivery, or breast milk. The worry is only for my child. I don't want my son to be worried about something he doesn't know."*

### Fear and distress in day-to-day activities

Due to the physical and physiological changes taking place in women's bodies, going through pregnancy, giving birth, and the postpartum period can be difficult times for women with HIV affecting their day-to-day activities. Considering the multifaceted impact of HIV on these women, they may have a double burden (for themselves and their offspring) during pregnancy and postpartum. In this study, all the women reported that living with HIV influences their day-to-day lives in many ways. One of the participants discloses her difficulties in securing a job as follows.

"*Being HIV positive, I can't easily go work and earn a living; for example, to be hired in a restaurant, they require HIV test results in a 39-year-old woman who has been living with the virus for six years."*

A 40-year-old woman who has been living with the virus for 19 years states:

*"I'm now beginning to experience some mental distress and frustration-like symptoms. I also have experienced insomnia at night, especially after eleven pm these days; I also have to talk to myself at night. All of this trouble may have been caused by the fact that they didn't give me the medication I had been taking since the doctor told me to change out the medication I used to take and start second-generation medications."*

### HIV status self-disclosure dilemma

There are numerous adverse personal and social effects of HIV infection. In the current qualitative study, several participants described the difficulties in disclosing their HIV status, especially to their husbands. Even then, all the participants told their HIV status to their husbands. However, most participants did not disclose their HIV status to their social circle and the public. And yet, they strived that they had to tell their HIV status. The participants were concerned that revealing their HIV-positive status to the public would trigger assumptions that they had experienced negative social attitudes, fear of rejection, and fear of blame, and they feared the associated stigma and discrimination that would follow. This could lead to isolation since social discrimination frequently affects women with HIV and the entire family. Despite these difficulties, some women dared to come forward and reveal their HIV status to the public, even if they privately expected the worst reaction from society. In this study, the participants reported the main barriers to HIV self-disclosure to the public.

Unfavorable societal attitudes toward pregnant and postpartum women living with HIV and stigma and discrimination in society were obstacles to self-disclosure to the public. Participants expressed that they were aware of the unfavorable attitudes in society that would likely result in stigma and discrimination if they disclosed their HIV status to the public. A 34-year-old woman who has been living with the virus for 14 years reported:

*"My reason for not disclosing my HIV status is stigma and discrimination against people with HIV infection. Even some people think that HIV can be transmitted through clothes. Most people have not given up discrimination yet; I'm afraid of being isolated, rejected, and not accepted."*

A 32-year-old participant who has been living with HIV for eight years affirms her choice not to disclose her status to the public as:

*"I remained silent about my HIV status since informing people that I am HIV positive is useless because all that will only lead to rumors and gossip about me."*

### Challenges faced and coping mechanisms used by HIV-positive women

#### Challenges faced

The women in our study spoke about various difficulties they faced as people living with HIV, from when they discovered their initial HIV-positive test result to their most recent pregnancy, birth, and the postpartum period. The difficulties include unfavorable societal attitudes towards them, the presence of stigma and discrimination in society, lack of financial means to secure adequate food and transportation, difficulty in getting jobs for a living, difficulty entering into and maintaining a marriage, substance addiction, and uncertainty about their Health, and social isolation.

A participant who self-disclosed her HIV-positive status to the public reported:

*"Being HIV positive prevents me from getting into a marital relationship. Once you have the virus and everybody is aware of it, nobody wants to get married to you. The problem is worse if you are upfront and clear.* … *Some people ask if the baby was mine. There were also people who bet money and asked me if she was my daughter. Some people came right and questioned whether women with HIV could give birth… I sometimes question whether I am a woman because of this.… I had a terrible addiction when I was pregnant with the current child, but I gradually gave up,"* a 40-year-old woman, living with HIV for 19 years.

Another participant described her situation as follows:

*"My being HIV positive changed my husband's behavior. Before I discovered my status, we were getting along well, but he has become different since he found that I have the virus. He started spending the night outside, which he didn't do before, and his mood changed. It is not known what will happen in the future. What if I speak up when something is bothering me? Where am I going to take this baby? Falling on the street and living there is hard. So, I tell myself I will wait a little longer and see what the child has to say when he grows up, "*a 25-year-old woman living with HIV for two years.

Some women struggled to pay even for transportation to the hospital. One of the participants, for instance, disclosed:

*"My husband does not help me in any way. I had to walk the entire distance to the hospital to take medicine for myself and these twins since I was running out of taxi money," a 36-year-old woman living with HIV for 11 years.*

A participant who self-disclosed her HIV-positive status to the public reported:

*"People are afraid of HIV these days, and discrimination exists. People were frightened to touch babies born from HIV-positive women because they believed they would contract a virus from touching them. Discrimination exists even against children and babies," a 40-year-old woman living with HIV for 19 years.*

### Coping mechanisms used for the challenges encountered

Having HIV has numerous personal and social repercussions. The study's findings revealed that the participants all employed various coping strategies to overcome their many challenges. The main challenges started when they discovered their first HIV-positive results, along with difficulties related to pregnancy, labor, postpartum, and their daily lives. Most women primarily used various forms of social support provided from multiple levels as coping strategies. However, some women used their spirituality as a means of self-acceptance. The remaining women were able to manage the challenges faced in the peripartum period using ignorance, confrontation, or isolation to concentrate on their futures as mothers.

### Social support

Support from significant others, friends, and professionals were one of the primary sources of coping mechanisms reported by women living with HIV. Most participants said that family support was their primary source of coping mechanisms when they first found their HIV-positive status. The participants received a range of support from various sources, primarily emotional and social support from partners, family, and friends on the interpersonal level and HIV-related healthcare delivery, along with excellent counseling and reassurance and system support on the organizational level by the healthcare providers. A 32-year-old participant who has been living with HIV for 12 years now reported having received significant support from her family:

*"My family members were there to support me when I got my first HIV-positive test results; they were the ones who got me evaluated because I was ill, and they were the ones who helped me to calm down."*

Another participant who received significant support from her husband revealed:

*"My husband was by my side when I got my first HIV-positive test results. He helped me a lot to get over it. I was comforted by that… After I gave birth to the current child, my husband supported me a lot,"* a 32-year-old woman living with the virus for eight years*.’’*

A 39-year-old participant who has been living with HIV for six years underlines friendship support.

*"I was in a desperate state when I discovered that I had HIV infection. A friend who revealed her HIV status to me and assured me that she was taking antiretroviral therapy (ART) and could lead a normal life greatly comfort me at that time."*

### Spirituality

Some women admitted that they had used their spirituality as a coping mechanism at various periods in their lives because they saw it to embrace themselves and their HIV status. The following anecdotes from two participants demonstrate spirituality employed as a coping strategy.

*"I reassure myself. I was praying and begging St. Virgin Merry to give me the strength to cope with what I faced. God, my lord, helped me to compose myself,"* a 36-year-old woman living with HIV for 11 years.

*"I found out that society has a negative attitude towards mothers living with HIV, but I was able to cope with God's help. I think I am also strong,"* a 40-year-old woman living with HIV for 19 years.

### Ignorance

Some participants used ignorance as a coping mechanism for their challenges. For instance, a 32-year-old woman who has been living with HIV for 12 years stated that the degree of ignorance she employed as a coping mechanism revealed:

*"I came across society's unfavorable attitude towards mothers living with HIV, but I don't give that much attention. My only priority is getting an HIV-free child."*

### Confrontation

Some women, especially those whom self-disclosed to the public, used confrontation as a coping mechanism. These women educated the community about the possibilities for women living with HIV to have an HIV-free child and other pertinent health information as a coping mechanism. A participant who self-disclosed her HIV-positive status to the public described that she constantly defended her by educating the public that those HIV-positive women can get an HIV free baby as far as she takes their ART appropriately and taking good care of the baby. She reported:

*“I constantly tell the community that women living with HIV can give birth to a healthy child. No matter what negative things society says about women living with HIV, the most important thing is that they can take care of themselves and have healthy children,"* a 38-year-old woman living with HIV for 11 years.

### Isolation

Some study participants have utilized isolation as a coping strategy to avoid the challenges faced as women living with HIV.

A participant who self-disclosed her HIV-positive status to the public described that society's attitude still hasn't changed:

*"Society does not have a good attitude toward people living with HIV. No one can hug a child born with HIV positive mother for this reason. I isolate myself from my neighbors to avoid their bad perception and gossip,"* a 39-year-old woman living with HIV for six years.

### Courage and commitment of HIV-positive women to get an HIV-free child

All the study participants expressed their determination and bravery to take the best possible care of their own, their husbands and their child's Health to prevent their child from contracting HIV, especially in the postpartum period.

All women who participated in this study acknowledged that they knew how HIV is transmitted from mother to child. As a result, they have exerted every effort to have HIV-free children by taking all necessary precautions, including taking their ART medications strictly as prescribed and using sharp objects at home and in their surroundings appropriately.

A 36-year-old participant who has been living with HIV for four years stated:

*"I carefully use my own possessions and keep sharp objects out of children's reach. All my children use their own tools, such as razors, toothbrushes, needles, and nail clippers. I constantly tell them to avoid sharp objects, such as needles or blades, falling on the ground. I also teach my children to avoid sharp things, even ones in the house. Additionally, my spouse and I both use condoms."*

Another participant stated her reasonable vigilance not to transmit the virus says:

*"I want to live responsibly for my children; I want them to be protected as they grow up. I care very much for all my children. I make sure to clean them, make sure they don't encounter anything that belongs to me, and take extra care while I'm menstruating, at the very least. I give each of them a razor and a toothbrush as a precaution,"* a 32-year-old woman living with HIV for seven years.

### Courage and commitment of HIV-positive women to prevent husbands from contracting HIV

Some participants in the current study revealed that their husbands are HIV-negative and are also aware of their wives' HIV-positive status. These women affirmed their commitment to preventing their husbands from contracting HIV using several preventative measures, including regular condom use and strict adherence to ART regimens. For instance, what one of such participants revealed is a case in point.

*"Since my husband is HIV negative, I always take extra precautions to avoid contaminating him, and as a result, we always use condoms when we have sexual contact, along with other things, we utilize our personal toothbrush, etc.," a 36-year old woman 11 years living with HIV."*

### Healthcare providers and HIV-positive women's interaction

All the women who participated in this study reported receiving proper medical treatment and care during pregnancy and in the postpartum period with good interaction with health professionals. For instance, a 36-year-old woman who has been living with the virus for four years stated her healthcare impression as follows:

*"I have got excellent health care in all phases during pregnancy, delivery, and in the follow-up period with excellent interaction with the health care providers.*"

Another participant perceived the support she received from the health care providers as:

"They gave me much help. They take good care of me when I come in. Everything is fine," a 32-year-old woman living with HIV for eight years.

A 34-year-old woman who has had the virus for over 14 years has a positive impression.

*"They helped me a great deal. It's just great. They have advised me to be strong to safeguard my health and the Health and future of my baby."*

### Solution from their voices

The study participants' voice reflects not only what happened and how they got through, but it also includes what could be done to make the life of women with HIV-Positive diagnoses easy postpartum. Those suggestions include changes in societal attitudes towards HIV-virus and creating societal awareness using social media; one of the participants, for example, says:

*"I do want to point out that society's attitude on HIV hasn't changed yet and that they continue to blame individuals living with the virus. I want the community to view HIV as one of any chronic illnesses," a 36-year-old woman living with HIV for over four years.*

Another suggestion voiced by the participants was that the role of the media has been deteriorating from where it was in the past. A 38-year-old participant who has had the virus for the last 11 years underlines:

*"In the past, when HIV was viewed as a horrible illness, it was announced in the media that women who are HIV positive could give birth to an HIV-free child. However, such topics are no longer covered by the media. I don't think that many people are concerned about HIV these days. Provided the media portrays it as possible for an HIV-positive mother to give birth to a healthy child if she took the necessary precautions, it is still possible to deal with it."*

A 32-year-old participant who has had the virus for over eight years strongly advises the due care mothers with HIV need to take to have an HIV-free child.

*"My advice to women living with HIV is to be very careful and take extra care to minimize contamination when raising children. I believe there is no greater satisfaction than giving birth to and raising an HIV-free child."*

It has been realized that HIV is treatable. However, it is not yet curable. Nonetheless, the younger generation is not serious about understanding this reality. Participants of the present study have forwarded serious recommendations on this issue based on their observations. A 34-year-old woman, for instance, pinpointed the following.

*"Although HIV is a silent weapon, it is not emphasized in today’s world. Young people do not take it seriously. The youth typically pretend there is no virus after midnight and question why the virus wouldn't appear dressed as a woman, and they don't take any care. If you see the young people here, they get drunk at night; chew Khat, you see a dream in every brothel and liquor store; it isn't good. There is no country without youth. We must act to protect our generation. Sadly, the government is not addressing this. Attention should be paid. HIV should be given priority."*

## Discussion

This study aimed to explore the lived experience of pregnancy and motherhood among women living with HIV. The findings depict the experience of pregnancy and motherhood after receiving an HIV diagnosis. Moreover, the results reveal important new information about the "lived experience" of HIV-positive women during pregnancy and postpartum from an Ethiopian perspective.

Because HIV affects every aspect of the participants' lives, so going through pregnancy and postpartum as mentioned in their narratives while living with HIV is challenging. The findings demonstrated that HIV-positive women's lived experiences are characterized by fear and distress related to maternal and child Health, HIV status self-disclosure dilemma, courage and commitment of HIV-positive women to prevent HIV challenges faced, and coping mechanisms used by HIV-positive women, health care provider's and HIV positive women interactions and solution from their voices.

### Fear and distress upon HIV diagnosis

Women participated in this study learned their HIV positive sero-status for the first time under various conditions, such as during pregnancy, labor, voluntary counseling, and testing (VCT), and when they went to health institutions to seek medical care. Following the HIV diagnosis, the participants experienced varied emotional reactions of fear and distress upon HIV diagnosis which was one of the themes identified from participant’s narratives. The test results were unanticipated and shocking for most women, though some were somewhat prepared for it due to risky behaviors. The emotional responses felt by women upon receiving an HIV diagnosis have been described in several types of research, which is consistent with our findings [9, 28, 42, 43]. This may be because HIV infection is an incurable illness, as well as the stigma, discrimination, and other forms of social and cultural embarrassment.

### Fear and distress during pregnancy, labor and in the postpartum period

Although all the participants were on ART, they had varying degrees of adverse emotional reactions related to the possibility that HIV could be transmitted from mother to child. Emotional responses of fear, distress, and uncertainty during pregnancy and postpartum related to maternal and child Health were one of the most frequently mentioned descriptions of the participants. The participants claimed that throughout their pregnancies, during the postpartum period, and up until the baby's HIV status was determined, they experienced fear, distress, and uncertainty regarding the health and HIV status of the child. The fear of passing the virus on to the child was a prevalent women's narrative. They claimed they were unsure of the child's HIV status because all the mothers breastfed their children. The participants also reported that their uncertainty went beyond physical discomfort; they also experienced sadness and shame over getting HIV and endangering their children's lives. However, they all believed that the risk might be reduced if the women demonstrated good adherence to antiretroviral medication and other safety measures during pregnancy and postpartum. Our results are consistent with other studies carried out elsewhere, which shown that HIV-positive women experienced negative emotions, including dread, anxiety, and guilt throughout pregnancy and in the postpartum period, associated with fear of passing the infection to the baby and having an HIV-positive child [13, 16, 21, 28, 32].

### HIV status self-disclosure dilemma

HIV status disclosure, defined as telling someone about one's HIV status directly or indirectly, can be challenging for women and is still a cause for concern [44, 45]. It usually is better to disclose an HIV-positive test since it gives everyone more time to adjust to the patient's disease. Moreover, it lessens the stress they could experience from keeping their condition private, enhances their chance of receiving more excellent social support, and helps them live effectively with the virus, including by implementing HIV prevention measures [29, 46].

HIV status self-disclosure is essential for women during pregnancy and the early postpartum period since disclosure is vital for the uptake and maintaining the usage of PMTCT services by encouraging treatment compliance [47]. In this study, several women described the difficulties in disclosing their HIV status even though; they all told their HIV status to their respective husbands. However, most participants did not disclose their HIV status to their social circle or the public. The participants in this study reported several obstacles to HIV self-disclosure to the public, including fear of rejection, blame, negative social attitudes, and a fear of the stigma and discrimination that would follow. These obstacles could result in isolation because social discrimination frequently affects women and the entire family. The complexity of how HIV-related stigma and discrimination influenced participants' desire to integrate into their social networks is shown through participant stories.

The stigma and discrimination associated with HIV continue to harm many people globally [48]. Studies have revealed that the stigma associated with HIV/AIDS undoubtedly impacted women's psychological well-being. The stigma has a terrible implication for HIV-positive women because the individual with the virus is thought to have acquired it through immoral sexual behavior and is now spreading it to an innocent child [7]. The participants in the present study have made great efforts to keep their HIV status concealed from society to protect themselves from stigma. Our finding is consistent with studies done in different parts of the world where most participants had disclosed their HIV status to their spouses. Still, most women tried to keep their diagnosis a secret from their family, friends, and the community out of fear of gossip and to save family members from experiencing emotional pain [13, 28, 32, 44, 45, 49].

### Challenges faced and coping mechanisms used

The participants in the present study described a wide range of challenges they faced during their pregnancies and after giving birth. These include stigma, discrimination, an unfavorable social attitude toward them, a lack of resources to secure adequate food and transportation, difficulty finding and keeping a job, problems getting married and maintaining it, substance abuse, uncertainty about their Health, and social isolation. Previous studies also showed that HIV-positive women had various issues throughout pregnancy and the postpartum period [7, 29, 32, 48].

The present study revealed that participants utilized various coping mechanisms to lessen their challenges. Coping is defined as the use of ideas and behaviors or conscious and voluntary mobilization of acts to control stressful conditions both internally and externally [50]. Evidence of coping was seen when a woman tried to avoid encountering negative energy in her daily life that may emanate from her thoughts and her family, friends, and society. Research findings revealed that women who use appropriate coping mechanisms to deal with social and psychological difficulties are likely to have healthy pregnancies and postpartum periods [29, 51].

In the present study, most participants stated that social support was their primary source of coping techniques when they initially learned they were HIV positive, as well as during pregnancy and postpartum. The participants received various support from various sources, including partners, family, friends, and health care providers. Most participants reported that these supports were their primary energy source, positively influencing their pregnancy and postpartum. These findings are consistent with previous studies, which said that support obtained from the husband, family, friend, and healthcare provider is linked to lower levels of stress, better medication compliance, and a decreased risk of psychological distress, all of which improve the quality of life for HIV-positive women [13, 16, 32, 43, 51, 52].

Some of the participants in our study also used spirituality as a coping mechanism**.** The women acknowledged using their faith as a coping mechanism at various times. This gave them purpose and inner strength, and they saw spirituality to accept themselves and their HIV condition. Moreover, most participants stated that their faith in God allowed them to deal with uncertainty and despair linked with the risk of passing on HIV to their children. This is consistent with other studies in that religion and prayer had a significant role in shaping the lives of individuals with HIV as a source of spiritual support [42, 53]. Besides, studies found that religious affiliation is common among women living with HIV who held the belief that God oversaw every element of their lives and gave them the power to handle the challenges of pregnancy and the postpartum period, and also religious affiliation plays a significant role in medication compliance and participation in PMTCT cares [13, 21, 29, 54, 55].

Some of the participants adopted coping mechanisms at a personal level, such as isolation, confrontation, and ignorance, to protect themselves from stigma, discrimination, judgment as well as rumors about them. HIV-related stigma has a significant impact on people's actions about disclosing their HIV status to relatives, friends as well as to the public as evidenced by study participants' accounts of the challenges this has on their need to integrate into their social systems. HIV-related stigma studies also showed that women with HIV primarily used these coping mechanisms to avoid the stigma and discrimination associated with being HIV positive and society's negative attitudes toward people living with HIV. These factors could have been emotionally draining for the women and resulted in relationship boundaries that were too restrictive [16, 21, 29, 44, 48].

### Courage and commitment to HIV-positive women to get an HIV-free child

Even though they all experienced fear, distress, and uncertainty throughout pregnancy and in the postpartum period related to fear of HIV transmission to the child, all the participant put all their energy into maintaining optimal Health and have been curious and diligent in safeguarding their baby from contracting the virus. This was made possible by adopting various precautions, such as looking after the mothers' Health by adhering to ART medications. All the study participants clearly stated that keeping a positive maternal role and preventing children from contracting HIV were their top priorities. Our research agrees with some other studies, which illustrated that HIV-positive women were highly motivated to enhance their compliance with treatment regimens and other cares both during pregnancy and after giving birth to protect their children from acquiring the infection and maintaining their well-being [13, 21, 29, 44].

### Healthcare providers and HIV-positive women providers Interaction

All the participants in the present study reported that they received appropriate medical treatment and care during pregnancy and in the postpartum period. This helped them to get a positive pregnancy outcome. Moreover, the participant's interactions with health professionals were generally positive, with much appreciation given to their respective healthcare providers. Our finding is in line with past studies, where women enrolled in PMTCT clinics described health professionals as being kind and encouraging, which helped women cope with the difficulties faced during pregnancy and in the postpartum period [16, 29, 56]. These may be related to the fact that there is a clinic dedicated to HIV care and treatment and that PMTCT is receiving enough attention these days because MTCT is the primary source of new HIV infection among children. Moreover, similar to what we discovered in our research, other studies also showed that most women were able to put aside their worries about vertical transmission, and they become courageous and take the necessary prevention techniques to get an HIV-free child with the help of reliable healthcare professionals [29, 56].

## Conclusion and recommendation

Although PMTCT and related cares are available, women living with HIV still confront various psychosocial obstacles during pregnancy and in the postpartum period that is rarely addressed in antenatal clinics. The lived experience of HIV-positive women during pregnancy and the postpartum period is associated with fear and distress related to maternal and child Health, HIV status self-disclosure dilemma, courage, and commitment of HIV-positive women to prevent HIV challenges faced and coping mechanisms used by HIV-positive women, health care providers, and HIV positive women interaction.

The results of the current study have implications for healthcare professionals, program designers for healthcare, and other stakeholders involved in the fight against HIV to be aware of the experiences of HIV-positive women during pregnancy and in the postpartum period to offer them specialized support and all-encompassing care so that they can have a healthy pregnancy and raise their children with a minimal uncertainty. Besides, health professionals must become familiar with the unique requirements from the lived experiences of pregnant HIV-positive women to support them as they take steps to maximize the protection of their child from contracting HIV and improve their mothering roles. In addition to providing for women’s needs in a patient-centered manner, it is essential to understand the circumstances of women's lives, their relationships with others, and their decision-making processes. Healthcare professionals and other responsible bodies working with women living with HIV should encourage and support these mothers to appraise and promote their commitment to protecting their children from acquiring HIV infection and maintaining their Health to the maximum to raise their children.

Healthcare professionals who give services to WLWH throughout pregnancy may assist in reducing psychosocial difficulties and psychological discomfort by supporting, promoting, and facilitating the use of the positive coping mechanisms that women are already employing; these initiatives include routinely checking for psychological distress in the HIV clinic and referring them for evaluation and treatment of their mental Health.

Community members, partners, health care providers, family members, and the larger structural environment, including policymakers, funders, and program implementers, should be involved and work together to empower communities through which WLWH can benefit to assist WLWH in overcoming the difficulties they face.

For researchers, we recommend they explore psychosocial challenges that cause women to isolate themselves from their social circles since this might weaken women's ability to engage in HIV care and adhere to ART. These challenges should then be addressed at the family and community levels.

### Limitations

Although the findings of this qualitative study added to the body of knowledge regarding the lived experience of HIV-positive women during pregnancy and in the postpartum period, it could have some limitations. One of the limitations could be not including pregnant women in the current study. We just tried to explore the lived experience of both pregnancy and the postpartum period by recruiting the women in their postpartum period. If it can be regarded as a limitation, we did not utilize any software to help with the data management coding process; instead, we carefully conducted the coding of the data manually. Since the topic under study was a private matter for the study participants, only the lead investigator (a nurse and reproductive health expert) performed the interview.

## Supplementary Information

**Additional file 1.**

## Data Availability

The corresponding author can share data when reasonable requests emerge.

## Abbreviations

AIDS: Acquired Immunodeficiency Syndrome
ANC: Anti Natal Care
AOR: Adjusted Odds Ratio
ART: Antiretroviral Treatment
CD4: A cluster of differentiation
cART: Combination antiretroviral therapy
HIV: Human Immune Deficiency Virus
IDIs: In-depth interviews
MTCT: Mother-to-child transmission
PMTCT: Prevention of mother-to-child transmission
PLWHA: People living with HIV and AIDS
PNC: Postnatal care
UNAIDS: The Joint United Nations Programme on HIV/AIDS
UNFPA: The United Nations Population Fund
WLWH: Women living with HIV
