# Supplementary material for: Women's voices: the lived experience of HIV-positive women in the postpartum period at the University of Gondar comprehensive specialized referral hospital, Ethiopia: a phenomenological study
Source: BMC Womens Health. 2023 Jun 21;23:325. doi: 10.1186/s12905-023-02470-5 (PMC10283323; doi:10.1186/s12905-023-02470-5)
Supplement: Supplementary file 1 — Additional file 1. [file 12905_2023_2470_MOESM1_ESM.docx]

**In-depth interview guide for lived experience of postpartum women**

**Part I: Socio demographic characteristics**

1. Age __________________
2. Place of residence ______________
3. Marital status _________________
4. Occupation _________________
5. Educational status___________________
6. Was the current pregnancy planned? ________________
7. Time between the current birth and the previous birth, if it is not the first birth ____
8. How many weeks passed since you gave birth to the current child? ________
9. What was the sero-status of your husband/partner ______________

Part II: HIV infection related

1. When did you know that you are living with HIV?
2. For how many years you are living with HIV?
3. In what circumstances did you know that you have HIV?
4. How did you respond when you heard that you were HIV positive?
5. Can you tell me how you managed yourself or cope with the situation when you heard about your HIV status for the first time?
6. Who helped you to get out of the situation?
7. How long you are on ART?
8. Have you disclosed your sero status to anyone?
9. If yes, for whom you told about your HIV status?
10. If you did not yet disclosed your sero status to important others, could you tell me the reason?

Part III: HIV, pregnancy and postpartum

1. What it feels to be pregnant and in the postpartum period as an HIV sero-positive women?
2. Could you please tell me how your HIV sero status affected your life?
3. Could you tell me about any problems that you encountered because of your HIV sero-status?

- Before you become pregnant with the current pregnancy.
- During the current pregnancy
- After the recent birth (postpartum)

1. How did you manage or cope the problems you encountered?
2. What do you think about the attitudes of the community towards you having a baby while HIV sero-positive? Could you tell me your experience in this regards?
3. How did you manage or cope the challenges faced from the community?
4. Could you describe how you see yourself and your future as a mother with positive sero-status?
5. Tell me about your experience with the healthcare providers during pregnancy, delivery or postpartum.
6. Have you faced any challenges related to HIV treatment (ART) and related care during pregnancy and postpartum period? Tell me about the challenges?
7. Have you been feeding your breast milk since birth?
8. Do you want to add anything else?
